# Cognitive Structuring and Its Cognitive-Motivational Determinants as an Explanatory Framework of the Fear-Then-Relief Social Influence Strategy

**DOI:** 10.3389/fpsyg.2017.00114

**Published:** 2017-01-30

**Authors:** Dariusz Dolinski, Barbara Dolinska, Yoram Bar-Tal

**Affiliations:** ^1^Faculty of Psychology in Wroclaw, SWPS University of Social Sciences and HumanitiesWroclaw, Poland; ^2^Faculty of Social Sciences, Opole UniversityOpole, Poland; ^3^Sackler Faculty of Medicine, Tel-Aviv UniversityTel-Aviv, Israel

**Keywords:** compliance, social influence, fear-then-relief, cognitive structuring

## Abstract

According to the fear-then-relief technique of social influence, people who experience anxiety whose source is abruptly withdrawn usually respond positively to various requests and commands addressed to them. This effect is usually explained by the fact that fear invokes a specific program of action, and that when the source of this emotion is suddenly and unexpectedly removed, the program is no longer operative, but the person has not yet invoked a new program. This specific state of disorientation makes compliance more likely. In this paper, an alternative explanation of the fear-then-relief effect is offered. It is assumed that the rapid change of emotions is associated with feelings of uncertainty and confusion. The positive response to the request is a form of coping with uncertainty. In line with this reasoning, while individuals with a high need for closure (NFC) should comply with a request after a fear-then-relief situation, low NFC individuals who are less threatened by uncertainty should not. This assumption was confirmed in the experiment.

## Introduction

The literature on persuasion and compliance provides descriptions of various procedures increasing the likelihood of compliance (see: Cialdini, 2001; Pratkanis, 2007; Dolinski, 2016; O’Keefe, 2016 for review). One such technique is the fear-then-relief strategy. In many experiments, it has been shown that when people experience a source of fear that is then rapidly removed, they are more likely to comply with a subsequent request. For example, in one set of studies participants were led to believe they had received a parking ticket or were caught jaywalking, which then turned out to be a false alarm. The ticket was in fact an advert for a hair formula, while the person blowing a whistle on the jaywalker was not the expected police officer, but rather a smiling jokester (Dolinski and Nawrat, 1998). In such cases, people were more likely to comply with a request to fill out a burdensome questionnaire or make a donation to an orphanage. How can the mechanism of compliance in such situations be understood? This finding is usually explained by noting that the state of sudden and unexpected relief from fear produces a temporal mindlessness (Dolinski, 2001, 2016). This interpretation is in accordance with the results of the experiment by Dolinski and Nawrat (1998), inspired by a study conducted by Langer et al. (1978). The participants were individuals crossing the street where it was not allowed. In one condition the fact was just recorded, while in the second the participants heard a police whistle (produced by the experimenter) as they crossed the street. Next, each participant was approached by a confederate asking for a donation. As in the original study by Langer et al. (1978), he formulated either the request only, the request with a real justification, or the request with a placebic justification. The results showed that in the control condition people usually behaved in a thoughtful manner. They hardly ever decided to give a donation when the request was not accompanied by any justification, nor when the justification was placebic, and more often made a donation when it was explained to them who was collecting the money and for what purpose. The participants in the fear-then-relief conditions reacted differently. In this case, it was enough to have any kind of justification at all for participants to be more likely to reach into their wallets when compared to conditions in which the request was made without accompanying justification. It also turned out that under fear-then-relief condition, participants approached with unusual messages (e.g., request with placebic justification or without any justification) hardly ever asked any questions about the aim of the action or campaign and the organizer of it. This pattern of results is then quite congruent with the assumption that in a situation involving a sudden withdrawal of the sources of an emotion, mindlessness is caused by temporary deficits of cognitive resources. This, in turn, is because cognitive resources are directed elsewhere, such as turning off the fear-activated program that is no longer adequate and/or coping with the physiological consequences of the sudden drop of excitement. One may therefore say that when an individual is in such an unusual state, the cognitive system is busy recovering its own balance. This interpretation is supported by results obtained in several other experiments (e.g., Dolinski et al., 2002; Nawrat and Dolinski, 2007; Dolinska and Dolinski, 2014; Kaczmarek, 2014).

In the present paper, however, we would like to offer another cognitive-motivational framework for the fear-then-relief phenomenon, which is complementary rather than competitive to that presented above. It is based on the assumption that the rapid change of emotions is connected with feelings of uncertainty and confusion. The positive response to the request is a form of coping with uncertainty (see van den Bos, 2001, 2009). If this is the case, people less tolerant of ambiguity are more prone to respond with positive behavior. Such people are characterized by a high need for closure (NFC) (Kruglanski, 2013). NFC is defined as “an individual’s desire for a firm answer to a question and an aversion toward ambiguity” (Kruglanski and Webster, 1996, p. 264).

According to Kruglanski’s Lay epistemology theory (Kruglanski, 2013), NFC is one of the three epistemic motivations which affect people’s process of knowledge generating and validating. The theory suggests a two-stage process in which during the first stage the specific content (the hypothesis) is created. This stage is termed “seizing.” The second stage is devoted to validation of the cognitive content. The validation stage involves an examination of the consistency between the hypothesis and the person’s available knowledge, as well as of alternative explanations to consistent or inconsistent cognitive content generated by the individual. A central factor in this stage is the epistemic motivation of the individual. The epistemic motivation affects the validation process through two mechanisms: “freezing” and “unfreezing.” Epistemic freezing causes the individual to shorten the validation process and accept the hypothesis in question as correct. In contrast, epistemic unfreezing prolongs the validation process, which in turn extends the period during which the individual is exposed to uncertainty. NFC is an epistemic motivation which governs the “freezing-unfreezing” activity. When high, NFC increases the individual’s tendency to employ faster epistemic freezing and achieve faster certainty in the validity of the hypothesis. High NFC is also characterized by the use of “category based” processing (Fiske and Pavelchak, 1986; Brewer, 1988), non-systematic and heuristic processes. High-NFC individuals prefer to use holistic and rapid processing, crudely differentiated categories, black-and-white type solutions, and over-simplified dichotomizations. All these enable the individual to achieve the goal of avoiding uncertainty. In contrast, low NFC is associated with unfreezing. That is, low NFC is associated with a longer process of validation and extended period of uncertainty regarding the validity of the hypothesis in question. Low NFC is often associated with the use of “piecemeal” or “systematic processing,” which is manifested in vigilant behavior based on a systematic and effortful search for relevant information, its evaluation, and unbiased integration (Janis and Mann, 1977; Chaiken et al., 1989). It is important to note that NFC is often conceptualized as a dimension which, at its high pole, predisposes individuals to use cognitive structuring to achieve certainty. At its low pole, however, it is not associated with indifference or low motivation to achieve certainty, but with a high tendency toward piecemeal processes (Kruglanski and Webster, 1996). Finally, according to lay epistemology theory, NFC is both a trait-like characteristic, as well as situationally determined.

When applying the explanation of NFC to the fear-then-relief phenomenon, it can be suggested that compliance with the request frees individuals from the need to validate how they want to respond to the request. This in turn extends the uncertainty experienced in the situation. Therefore it is expected that participants’ NFC levels would moderate the effect of fear-then-relief. While individuals with high NFC will comply with a request after a fear-then-relief situation, low-NFC individuals who are less threatened by uncertainty will not.

## Materials and Methods

### Participants

A group of 120 university students (60 women, 60 men, aged 17–53 with *M* = 22.95; *SD* = 6.63) were randomly assigned to one of the two conditions: control and experimental (fear-then-relief).

### Procedure and Measures

In a laboratory room participants individually completed a scale devoted to measuring their NFC. We used the Polish version (Kossowska, 2003) of the NFC scale (Webster and Kruglanski, 1994). Respondents rated 32 items on a six-point scale (from 1 = completely disagree to 6 = completely agree). The reliability of the scale in the present study was high (Cronbach’s α = 0.82). The mean score for all items was calculated: *M* = 3.69; *SD* = 0.46. A higher mean score indicated a higher level of NCS.

When the scale was completed the experimenter thanked the participant, and at this very moment a female confederate entered the room saying “sorry for interrupting.” In the fear-then-relief condition she continued, asking the participant “Haven’t you lost your wallet?” At this moment, participants impulsively checked their pockets or searched their bags. They also surveyed the wallet simultaneously presented by the confederate (the one chosen for the study was very original and unusual) and then stated that it was not they who had lost the wallet. The confederate thus continued: “Oh, I’ll have to take it to the University Information Desk then… but as we are already talking… I am a member of a students’ committee organizing the celebrations of the 20th anniversary of our University. Would you agree to help us organize the events?” If the answer was positive, the confederate continued: “how many hours of activity do you declare, more or less?”

Two of the participants (one female and one male) did not respond in accordance with the assumed scenario, and in response to the confederate’s question “Haven’t you lost your wallet?” calmly responded that they had not, without betraying any sign of disquiet. In addition, during the post-experiment debriefing two other individuals (men) declared their suspicions that the episode with the wallet was not an accident, but rather an element in the experiment. The results of all these participants were excluded and four additional individuals were included in the study.

In the control condition the confederate entered the room, excused herself for causing the interruption and asked the participant for assistance in organizing events during the anniversary celebrations.

The participant’s consent to help in the organization of the anniversary celebrations and the number of hours of activity declared were treated as dependent variables.

At the very beginning of the experiment, just after arriving at the laboratory, study participants signed a declaration stating that they expressed their informed consent to participation in a psychological experiment which would measure certain individual character traits, as well as reactions to various events (because of the necessity of inducing in the participants a state of unexpected fear followed by relief, it was not possible to give them full and detailed information about the experimental procedure). Immediately after the experiment, each study participant went through a thorough debriefing process. None of them expressed any reservations concerning the course of the experiment. The design and the experiment conditions for the study were approved by the local University of Social Science and Humanities Ethics Committee in accordance with the Helsinki Declaration.

## Results

To test the hypothesis on compliance, we performed a logistic regression in two steps. In the first step, we introduced the two independent variables (NFC and manipulation), and in the second step the interaction between the two variables. **Table 1** shows that in the first step only the manipulation had a significant effect on compliance. The interaction in the second step was of marginal significance. In addition, we performed a regression analysis with the same independent variables on the second dependent variable (number of hours). **Table 2** shows that in the first step only the manipulation (fear-then-relief vs. control condition) achieved significance. In the second step, the interaction term contributed significantly to the explanation of the dependent variable. To further explore the source of the interaction two simple slopes were calculated for high and low NFC individuals (one SD above and below the mean). The results show that while for low-NFC individuals the regression coefficient is not significant (*b* = 0.11, *SE* = 0.56, *t* = 0.21, *p* = 0.83, LLCI = -0.99, ULCI = 1.22), it is significant for high-NFC individuals (*b* = 2.14, *SE* = 0.55, *t* = 3.84, *p* = 0.002, LLCI = 1.03, ULCI = 3.24). Thus, the research hypothesis is confirmed. The effect of fear-then-relief on low-NFC individuals was lower than on high-NFC individuals. In fact, the effect of fear-then-relief exists only in the case of high-NFC individuals.

**Table 1:** Logistic regression of the moderating effect of need for closure (NFC) on the relationship between manipulation of fear-then-relief state and binomial measure of compliance.

	*B*	*SE*	Wald	Cox & Snell *R^2^*	Sig.
Step 1				0.13	
NFC	-0.03	0.21	0.2		0.88
Conditions	0.83	0.22	14.06		0.00
Step 2				0.15	
NFC × Conditions	0.42	0.24	3.10		0.08

**Table 2:** Regression analysis of the moderating effect of NFC on the relationship between manipulation of fear-then-relief state and the interval measure of compliance.

	*B*	*SE*	β	*T*	Sig.
Step 1					
NFC	0.10	0.40	0.02	0.24	0.81
Conditions	1.13	0.40	0.25	2.80	0.01
Step 2					
NFC × Conditions	1.01	0.40	0.22	2.56	0.01

## Discussion

### Theoretical Implications

In our experiment, we demonstrated the existence of individual differences in susceptibility to the “fear-then-relief” technique. In respect of compliance measured on an interval scale (declared number of hours of activity on behalf of the University), the technique was successful in respect of individuals characterized by a high need for cognitive closure, while ineffective toward those with a low need for cognitive closure. This effect expands knowledge on the efficacy of social influence techniques, and particularly that of fear-then-relief. It also constitutes a complementary theoretical interpretation of the effectiveness of that technique compared to the “classic” one which assumes that the feeling of sudden relief induces a state of mindlessness (Dolinski, 2001). The results we have generated allow us to assume that in a condition of relief following the experience of fear, people are motivated to reduce uncertainty. The research presented in this article corresponds well with other data on the role played by cognitive structuring in various social influence processes. There is empirical data showing that a high need for cognitive closure is conducive to conformism (Calogero et al., 2009; Chao et al., 2010), susceptibility to the disrupt-then-reframe technique (Kardes et al., 2007), and to persuasion in conditions of absence of an initial informational base for an opinion (Kruglanski et al., 1993). Individuals characterized by high NFC are also more susceptible to depletion of cognitive resources than those with low NFC in conditions involving resisting the influence of an authority figure (Damen et al., 2014).

### Practical Implications

From the perspective of the practical application of fear-then-relief, we may posit the careful hypothesis that it should be particularly successful in situations involving conditions of uncertainty and confusion (this carefulness results from the fact that in our study we did not apply situational stimulation of NFC, and we treated this construct as a stable individual trait). It is also worth observing that practitioners of social influence usually do not apply individual techniques, but a chain of various methods, and they modify their selection of persuasive strategies based on the development of the situation (e.g., Goldman, 1986; Howard, 1995). It would therefore seem that if the fear-then-relief fails in respect of a particular person, this will be someone characterized by a low NFC rather than someone with a high NFC. Practitioners of social influence should thus apply measures that are appropriate for people with low NFC (e.g., present them a large amount of new and complex information – see: Luttrell et al., 2014 for review).

### Limitations

A few limitations associated with our empirical determinations should be emphasized. Firstly, the conclusion on the role played by need for cognitive closure in compliance with fear-then-relief is based on one study. Secondly, in our study need for cognition was treated as a personality factor. It is not certain whether an analogical structure of results would be achieved by treating need for cognitive closure as a factor of a situational nature. Thirdly, the role of need for cognitive closure was only observed when compliance with the request was treated as an interval variable (number of declared hours of work for the University). For the dichotomous variable (do you agree to participate in organizing a celebration for the University’s 20th anniversary?) only the main fear-then-relief effect was clearly noted, and the interaction of this factor with NFC did not achieve statistical significance.

### Future Directions

Although our results are congruent with the assumption that uncertainty is particularly aversive for people characterized by high need for cognitive closure, and this is precisely why they are susceptible to the fear-then-relief technique, we did not directly examine the mechanism itself. Future experiments should thus aim at exploring the mediational role of experiencing uncertainty. In other studies devoted to the role played by cognitive structuring processes in the fear-then-relief technique, we plan to examine whether an analogical pattern of dependencies is obtained when we manipulate the NFC (rather than measure it with a survey as in the present study). We also intend to examine the role played by another element of cognitive structuring, namely, ability to achieve cognitive structure. From the theoretical perspective, we may expect that differentiated ability should also lead to differentiated reactions to the request that appears when the individual first experiences an unexpected fear, and then unexpected relief (and thus in an unclear and unexpected situation).

## Ethics Statement

At the very beginning of the experiment, just after arriving at the laboratory, study participants signed a declaration stating that they expressed their informed consent to participation in a psychological experiment which would measure certain individual character traits, as well as reactions to various events (because of the necessity of inducing in the participants a state of unexpected fear followed by relief, it was not possible to give them full and detailed information about the experimental procedure). Immediately after the experiment, each study participant went through a thorough debriefing process. None of them expressed any reservations concerning the course of the experiment. The design and the experiment conditions for the study were approved by the local University of Social Science and Humanities Ethics Committee in accordance with the Helsinki Declaration.

## Author Contributions

DD – general idea of the research, design of the experiment, and manuscript preparation. BD – general idea of the research and manuscript preparation. YB-T – general idea of the research, data computing, and manuscript preparation.

## Conflict of Interest Statement

The authors declare that the research was conducted in the absence of any commercial or financial relationships that could be construed as a potential conflict of interest.
